# Disruption of Accumbens and Thalamic White Matter Connectivity Revealed by Diffusion Tensor Tractography in Young Men with Genetic Risk for Obesity

**DOI:** 10.3389/fnhum.2018.00075

**Published:** 2018-02-22

**Authors:** Gaia Olivo, Francesco Latini, Lyle Wiemerslage, Elna-Marie Larsson, Helgi B. Schiöth

**Affiliations:** ^1^Functional Pharmacology, Department of Neuroscience, Uppsala University, Uppsala, Sweden; ^2^Neurosurgery, Department of Neuroscience, Uppsala University, Uppsala, Sweden; ^3^Neuroradiology, Department of Surgical Sciences, Radiology, Uppsala University, Uppsala, Sweden

**Keywords:** FTO, DTI, tractography, MRI, accumbens, thalamus, obesity, white matter

## Abstract

**Background**: Neurovascular coupling is associated with white matter (WM) structural integrity, and it is regulated by specific subtypes of dopaminergic receptors. An altered activity of such receptors, highly expressed in reward-related regions, has been reported in carriers of obesity-risk alleles of the fat mass and obesity associated (*FTO*) gene. Among the reward-related regions, the thalamus and the nucleus accumbens are particularly vulnerable to blood pressure dysregulation due to their peculiar anatomo-vascular characteristics, and have been consistently reported to be altered in early-stage obesity. We have thus hypothesized that a disruption in thalamus and nucleus accumbens WM microstructure, possibly on neurovascular basis, could potentially be a predisposing factor underlying the enhanced risk for obesity in the risk-allele carriers.

**Methods**: We have tested WM integrity in 21 male participants genotyped on the *FTO* risk single nucleotide polymorphisms (SNP) rs9939609, through a deterministic tractography analysis. Only homozygous participants (9 AA, 12 TT) were included. 11 tracts were selected and categorized as following according to our hypothesis: “risk tracts”, “obesity-associated tracts”, and a control tract (forcpes major). We investigated whether an association existed between genotype, body mass index (BMI) and WM microstructural integrity in the “risk-tracts” (anterior thalamic radiation and accumbofrontal fasciculus) compared to other tracts. Moreover, we explored whether WM diffusivity could be related to specific personality traits in terms of punishment and reward sensitivity, as measure by the BIS/BAS questionnaire.

**Results**: An effect of the genotype and an interaction effect of genotype and BMI were detected on the fractional anisotropy (FA) of the “risk tracts”. Correlations between WM diffusivity parameters and measures of punishment and reward sensitivity were also detected in many WM tracts of both networks.

**Conclusions**: A disruption of the structural connectivity from the nucleus accumbens and the thalamus might occur early in carriers of the *FTO* AA risk-allele, and possibly act as a predisposing factor to the development of obesity.

## Introduction

The genetic risk for obesity has been linked to more than 300 single nucleotide polymorphisms (SNP; Goodarzi, 2017). The fat mass and obesity associated (*FTO*) gene (Yang et al., 2012; Loos and Yeo, 2014), one of the first obesity-genes ever identified, remains nonetheless one of the most strongly associated with obesity (Goodarzi, 2017). The association between risk-variants of the *FTO* gene and obesity seems not to be mediated by peripheral factors such as a dysfunctional metabolism (Cecil et al., 2008; Speakman et al., 2008), but rather by increased dietary intake and unhealthy eating behaviors (Brunkwall et al., 2013), probably linked to an aberrant functioning of the reward network (Hess et al., 2013; Sevgi et al., 2015). The reward network encompasses several brain regions in the cortico-basal ganglia-thalamo-cortical loop (Yager et al., 2015), in which the nucleus accumbens (Camara et al., 2009) and thalamus (Yager et al., 2015) play a key role. It can be divided into different specialized sub-networks (Camara et al., 2009), and exerts several complex functions in human behavior, including reinforcement learning, novelty processing, decision making, incentive motivation, and addiction (Camara et al., 2009). Given its complexity, its activity is closely associated to that of other main networks, such as the salience network, the emotional arousal network and the executive network (Gupta et al., 2015). Brain structural and functional connectivity changes in the reward system, closely resembling those observed in addiction (Michaud et al., 2017), have also been reported in obesity (Marqués-Iturria et al., 2015; Blechert et al., 2016; Carnell et al., 2017; Papageorgiou et al., 2017; Verdejo-Román et al., 2017), affecting several tracts (e.g., the anterior thalamic radiation, accumbofrontal fasciculus, forceps minor (FMi), cingulum, superior longitudinal fasciculus, inferior fronto-occipital fasciculus (Cho et al., 2013; Marqués-Iturria et al., 2015; Kullmann et al., 2016; Nangunoori et al., 2016; Papageorgiou et al., 2017)) of the reward pathway (Sesack and Grace, 2010; Xu et al., 2012; Bracht et al., 2015; Yang et al., 2017). Only few studies, however, have investigated whether structural connectivity might be disrupted in people with genetic risk for obesity (Dennis et al., 2014; Spieker et al., 2015), reporting somewhat conflicting results.

The *FTO* gene modulates the activity of the midbrain reward circuitry by regulating the activity of the receptors D2 and D3 (Hess et al., 2013; Sevgi et al., 2015; Heni et al., 2016), providing a potential mechanism for the increased risk for obesity associated to the *FTO* risk-variants. In fact, an aberrant dopaminergic signaling in reward-related regions has been often reported in obesity (Frank, 2015), particularly through the hyposensitivity of receptors D2 and D3 (Frank, 2015). Moreover, dopaminergic receptors are involved in neurovascular coupling and pressure regulation (Perles-Barbacaru et al., 2011), which is associated with white matter (WM) structural integrity (Sorond et al., 2013; Chapman et al., 2015), and exerts an important role in neuroinflammatory processes (Soto et al., 2015; Wilhelm et al., 2017). The thalamus and the nucleus accumbens seem to be particularly vulnerable to blood pressure dysregulation, due their peculiar anatomo-vascular characteristics (Moody et al., 1990; Perles-Barbacaru et al., 2011; Iozzo, 2015). Accordingly, the gray matter (GM) volume of the nucleus accumbens has been reported to be lower in the at-risk AA carriers of the *FTO* SNP rs9939609 (de Groot et al., 2015).

We suggest that the *FTO* risk-allele might be associated with WM microstructural damage in specific reward-related regions, namely the thalamus and nucleus accumbens, particularly vulnerable to blood pressure dysregulation (Moody et al., 1990; Perles-Barbacaru et al., 2011; Iozzo, 2015). The subsequent WM disruption in regional connectivity might be a predisposing factor for the development of obesity. The disruption of other reward system tracts would then follow, depending on whether clinical obesity is developed.

To test our hypothesis, we have focused on 21 male participants genotyped for *FTO* SNP rs9939609. Variants of the SNP rs9939609 of the *FTO* gene are linked to an increased risk for obesity (Yang et al., 2012; Loos and Yeo, 2014), with the AA genotypes considered at-risk compared to the TT genotype (Frayling et al., 2007; Jacobsson et al., 2012; Sällman Almén et al., 2013). We have investigated whether an association existed between genotype, body mass index (BMI) and diffusivity parameters of WM, reflective of WM microstructural integrity, in the “risk tracts” (anterior thalamic radiation and accumbofrontal fasciculi) and the “obesity-associated tracts” (FMi, cingulum, superior longitudinal fasciculus, inferior fronto-occipital fasciculus). These tracts were selected for their role in reward and punishment sensitivity (Sesack and Grace, 2010; Xu et al., 2012; Bracht et al., 2015; Yang et al., 2017), and for their involvement in obesity (Cho et al., 2013; Marqués-Iturria et al., 2015; Kullmann et al., 2016; Nangunoori et al., 2016; Papageorgiou et al., 2017). Moreover, we explored whether WM diffusivity could be related to specific personality traits in terms of punishment and reward sensitivity, as measured by the BIS/BAS (Carver and White, 1994) questionnaire.

## Materials and Methods

### Participants

Prior to any experimental procedures, all participants gave written informed consent to the study which conformed to the Declaration of Helsinki and was approved by the Ethical Review Board of Uppsala, Sweden. Participants were 21 right-handed, northern-European males, with a mean age of 25 years (±2.0 years; range 20–28 years), recruited locally in Uppsala, Sweden by advertisement. Genotyping of the *FTO* single nucleotide polymorphism (SNP) rs9939609 was performed with a pre-designed Taqman single-nucleotide polymorphism genotyping assay (Applied Biosystems, Foster City, CA, USA) and an ABI7900 genetic analyzer with SDS 2.2 software at the Uppsala Genome Center^1^. The genotype call rate was 97.8%. Only homozygous participants were included in the study. Nine participants were homozygous for the risk A allele, 12 participants were homozygous for the non-risk T allele. The mean BMI of the sample was 25.2 Kg/m^2^ (±3.4 Kg/m^2^; range 20.4–32.9 Kg/m^2^). The demographic data of the participants are reported in Table 1.

**Table 1:** Demographics and neuropsychological scores of the participants.

	AA genotype (mean; SD)	TT genotype (mean; SD)
*Age (years)*	25 (± 2.6)	25 (± 1.6)
*BMI (Kg/m^2^)*	26.4 (± 3.7)	24.3 (± 3.0)
*BMI > 30 Kg/m^2^*	*N* = 2	*N* = 1
*BIS/BAS ratio*	2.1 (± 0.6)	2.1 (± 0.5)
*BAS Drive*	8.9 (± 1.8)	7.7 (± 2.4)
*BAS Fun Seeking*	13.1 (± 1.9)	11.3 (± 2.8)
*BAS Reward Responsiveness*	15.4 (± 2.3)	16.4 (± 1.8)
*BIS*	18.2 (± 3.0)	17.7 (± 4.1)

### Neuropsychological Testing

Clinical measures for punishment sensitivity and reward-seeking behavior were acquired through the Behavioral Inhibition and Activation Systems (BIS and BAS, respectively) questionnaires (Carver and White, 1994). The BIS is associated with behavioral withdrawal, punishment and unhealthy behavior (Carver and White, 1994; Voigt et al., 2009), while the BAS reflects impulsivity, approach behaviors and reward sensitivity (Carver and White, 1994). The questionnaire is composed of 24 items. Each item is represented by a statement, which the participants indicates how much he agrees or disagrees with on a four-point scale. The BIS includes only one scale, evaluating the reactions to the anticipation of punishment and anxiety. The BAS comprises three subscales: (1) the Drive scale is pertinent to the pursuit of desired goals; (2) the Fun Seeking scale evaluates the desire for new rewards and impulsivity; and (3) the Reward Responsiveness scale focuses on the positive reactions anticipating the rewards. The BIS/BAS ratio (BBr) was also calculated, which reflects the imbalance between the Activation and Inhibition system.

### DTI Acquisition

MRI scanning was performed with a Philips 3-Tesla (Achieva, Philips Healthcare, Best, Netherlands) using a 32-channel head coil. Diffusion tensor imaging (DTI) data were acquired using an echoplanar imaging sequence (TR: 6700 ms, TE: 77 ms, voxel size: 1.75 × 1.75 × 1.75 mm^3^, 1 b0, b value = 1000, 48 directions, 60 axial slices covering the whole brain). During the MRI study, the participants lay supine with the head fixed by straps and foam pads to minimize head movement.

### DTI Pre-processing

All pre-processing steps were carried out in FMRIB Software Library (FSL, provided in the public domain by the Oxford Centre for Functional Magnetic Resonance Imaging of the Brain; Jenkinson et al., 2012). DTI images were first corrected for eddy currents and head motion using the FMRIB’s Diffusion Toolbox (FDT) implemented in FSL, then brain images were extracted using the brain extraction tool (BET; Smith, 2002). All scans were visually inspected to check for artifacts and ensure good quality of the acquisition. The diffusion tensor model was then fitted at each voxel using Diffusion Toolkit^2^, obtaining fractional anisotropy (FA) and apparent diffusion coefficient (ADC) maps. FA measures the fraction of water molecules preferentially moving along the major axis of the axons, and reflects the overall integrity of the fiber (Alexander et al., 2007). ADC reflects the impedance of water molecules, with higher ADC values associated to higher space between myelin sheets layers (Alexander et al., 2007). The maximum turning angle was set at 35° (Diffusion Toolkit default) and minimum FA was set at 0.1 (Soares et al., 2013) for DTI reconstruction.

### Tractography

The tractography analysis was performed with Trackvis^3^. Eleven WM tracts were selected *a priori* for tractography based on previous literature and manually identified: FMi (Papageorgiou et al., 2017), right and left anterior thalamic radiations (ATR; Kullmann et al., 2016; Papageorgiou et al., 2017), right and left cingulum (Papageorgiou et al., 2017), right and left accumbofrontal fasciculi (Cho et al., 2013; Marqués-Iturria et al., 2015; Nangunoori et al., 2016), right and left inferior longitudinal fascicule (IFOF; Papageorgiou et al., 2017), right and left superior longitudinal fasciculi (SLF; Kullmann et al., 2016; Papageorgiou et al., 2017). The ROIs were drawn according to Wakana et al. (2007), except for the accumbofrontal fasciculus, which was drawn based on Rigoard et al. (2011) and Vergani et al. (2016) studies. According to Wakana et al. (2007), the cingulum was split into a cingulate gyrus part, and a hippocampal part (CGH); the SLF was also divided in SLF, and temporal part of the SLF. In addition, the forceps major, which is not directly involved in reward, was selected as control tract, for a total of 16 selected tracts/subdivisions. FSL was used to extract the FA, ADC and volume of each tract.

### Networks Selection

**Figure 1:**
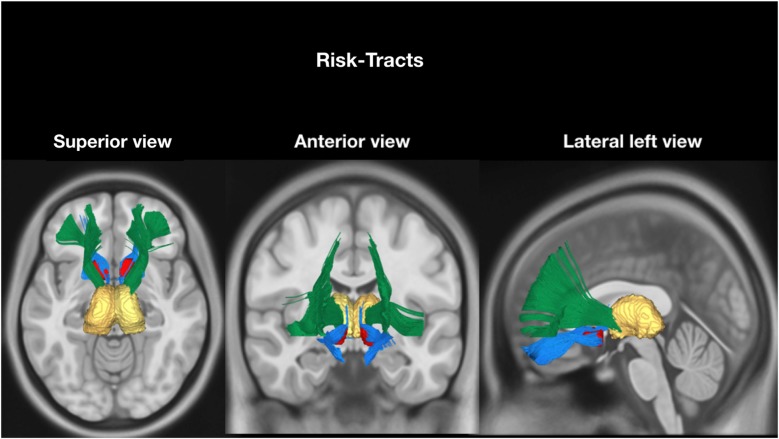
Tractographic reconstruction of “risk tracts” overlaid on a representative high resolution T1-weighted sequence slices from the Montreal Neurological Institute space. The previously acquired tracks (from Trackvis) and the MRI sequence were merged within the MNI space using DSI software (DSI Studio; freely downloadable at http://dsi-studio.labsolver.org/download-images) utilized for illustrative purpose only. The anatomical relationship of the two “risk tracts” bundles (Accumbens-Frontal, blue and Anterior Thalamic Radiation (green) with nucleus Accumbens (red) and thalamus (yellow) bilaterally, are displayed in axial (superior view), coronal (anterior view) and sagittal) lateral left). The nucleus accumbens and the thalamus were reconstructed within the MNI Space using Freesurfer atlas included in the DSI studio software.

**Figure 2:**
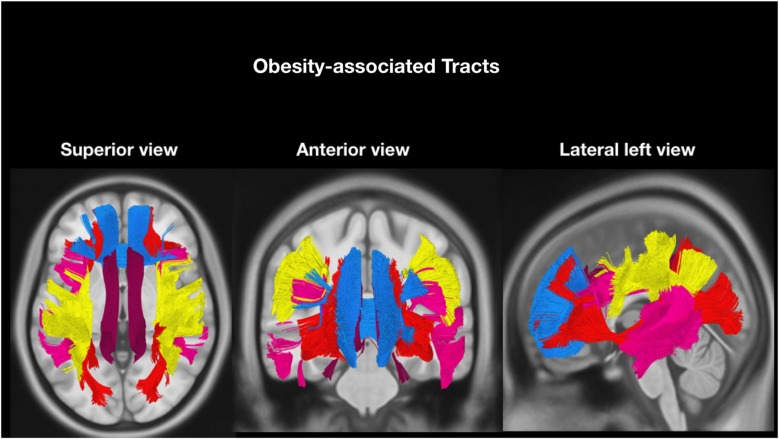
Tractographic reconstruction of “obesity-associated tracts” overlaid on a representative high resolution T1-weighted sequence slices from the Montreal Neurological Institute space. The previously acquired tracks (from Trackvis) and the MRI sequence were merged within the MNI space using DSI software (DSI Studio; freely downloadable at http://dsi-studio.labsolver.org/download-images) utilized for illustrative purpose only. The anatomical relationship of the bilateral “obesity-associated tracts” bundles (Arcuate fasciculus, pink; horizontal component of Superior longitudinal fasciculus, yellow; Inferior-fronto-occipital fasciculus, red; Cingulum, dark red and Forceps minor (FMi), blue) are displayed in axial (superior view), coronal (anterior view) and sagittal (lateral left view).

According to our hypothesis, the tracts were divided into “risk tracts”, comprising the accumbofrontalfasciculi and ATR (Figure 1), and “obesity-associated tracts”, comprising the cingulum (Ci) subdivisions, the SLF subdivisions, the IFOF and the FMi (Figure 2). This approach was chosen according to differences in vulnerability to pressure dysregulation based on different patterns of vascular architecture. Basal ganglia nuclei have been reported to be particularly vulnerable to pressure dysregulation compared to the other regions (Moody et al., 1990; Perles-Barbacaru et al., 2011; Iozzo, 2015) and therefore included into the suspected “risk stage” of WM damage. Because of differences in collateral blood supply and anastomotic compensation of peripheral cortico-sub-cortical regions Ci, SLF, IFOF and FMi were considered as “obesity-associated tracts” (Moody et al., 1990; Perles-Barbacaru et al., 2011; Iozzo, 2015). Mean FA and ADC of all tracts are reported in Table 2.

**Table 2:** Tracts characteristics.

	Fractional anisotropy (mean; SD)	Apparent diffusion coefficient (mean; SD) (×10^−3^ mm^2^/s)	Volume (mean; SD) (number of tracts)
Accumbofrontal Fasciculus, R	0.34; 0.03	0.80; 0.04	956; 191
Accumbofrontal Fasciculus, L	0.34; 0.03	0.82; 0.04	1062; 254
Anterior Thalamic Radiation, R	0.37; 0.02	0.80; 0.04	860; 398
Anterior Thalamic Radiation, L	0.37; 0.02	0.80; 0.04	849; 363
Cingulum, R (hippocampal part)	0.32; 0.03	0.85; 0.04	408; 152
Cingulum, L (hippocampal part)	0.32; 0.04	0.87; 0.04	472; 169
Cingulum, R (cingulate gyrus)	0.42; 0.04	0.74; 0.03	351; 183
Cingulum, L (cingulate gyrus)	0.42; 0.06	0.76; 0.04	457; 154
Forceps Major	0.48; 0.04	0.89; 0.09	1781; 495
Forceps Minor	0.45; 0.04	0.81; 0.04	2216; 754
Inferior Fronto-occipital Fasciculus, R	0.44; 0.02	0.80; 0.02	1479; 424
Inferior Fronto-occipital Fasciculus, L	0.45; 0.02	0.79; 0.02	1361; 562
Superior Longitudinal Fasciculus, R	0.39; 0.02	0.75; 0.02	1242; 497
Superior Longitudinal Fasciculus, L	0.40; 0.02	0.75; 0.02	1073; 470
Superior Longitudinal Fasciculus, R (temporal part)	0.43; 0.03	0.73; 0.03	442; 363
Superior Longitudinal Fasciculus, L (temporal part)	0.42; 0.03	0.75; 0.02	902; 365

### Statistical Analysis

All statistical analyses were performed with Statistical Package for Social Science (SPSS) v.24^4^. Prior to all analyses, the FA and ADC values of each tract were corrected for the volume of the tract through a linear regression analysis. The standardized residuals were then used for subsequent analyses. Multiple separate analyses of variance (ANOVA) analyses were first carried out to test for an effect of genotype and/or an effect of the interaction between genotype and BMI, in FA and ADC values of each tract separately. An interaction exists when the effect of one explanatory variable (i.e., genotype) on an outcome variable (i.e., FA or ADC) depends on the level or value of another explanatory variable (i.e., BMI). The threshold for significance was set at *p* < 0.003, to correct for multiple testing according to Bonferroni (0.05/16 tracts). Age was entered as nuisance covariate in the analysis.

To test our hypothesis, we then performed a multivariate analysis of variance (MANOVA) separately for the tracts included in the “risk” and “obesity-associated” network respectively. The FA and ADC values of the WM tracts were entered as dependent variables in different multivariate models respectively, and genotype and BMI were tested for main effects and interaction effect on the diffusivity parameters. Age was entered as nuisance covariate in all models. The model was also tested against the FA and ADC of the control tract (forceps major). The threshold for significance was set at *p* < 0.05.

The FA and ADC of the tracts included in each network were also tested for correlation with the psychological scores, through a series of univariate analyses of variance models. For the BIS/BAS questionnaire, the four subscales and the BBr were tested in five different models. The threshold for significance was set at *p* < 0.01, to allow for multiple testing correction according to Bonferroni’s approach (0.05/5). Age was again entered as nuisance covariate. In each model, the WM tracts comprising the network of interest were entered as independent variables.

## Results

### Difference in Diffusivity Parameters between Groups

No effect of genotype or interaction between genotype and BMI was found on the tracts FA when tested separately with the ANOVA analyses. An effect of the genotype and of the interaction between genotype and BMI was found on the ADC of the right accumbofrontal fasciculus (*p* < 0.022 and *p* < 0.016, respectively), not surviving the correction for multiple testing. An effect of the genotype and of the genotype*BMI interaction was also detected on the ADC of the right temporal part of the SLF (*p* < 0.028 and *p* < 0.026, respectively), not surviving the correction for multiple testing.

### Association between Genotype, BMI and Diffusivity Parameters of the “Risk Tracts”

At the MANOVA analyses, the FA values of the “risk tracts” were found to be significantly associated to genotype (*F* = 4.45, df = 13, *p* < 0.017) and to the interaction between genotype and BMI (*F* = 4.6, df = 13, *p* < 0.015), though not to BMI *per se*. In particular, the FA (i.e., standardized residuals after correction for tracts volume) of the left ATR and of the right accumbofrontal fasciculus was lower, while the FA of the right ATR and left accumbofrontal fasciculus was higher, in the risk-allele carriers. The “obesity-associated tracts” did not correlate either with genotype, BMI nor their interaction, supporting our hypothesis. No association was found between genotype, BMI and the FA of the forceps major (Table 3).

**Table 3:** Main effects and interaction between genotype, BMI and diffusivity parameters.

a. risk-network	Parameter	*F*	*η*^2^^‡^	Sig.
*Genotype*	FA	4.451	0.578	0.017*
*BMI*	FA	1.035	0.242	0.426
Genotype*BMI	FA	4.618	0.587	0.015*
*Genotype*	ADC	2.072	0.389	0.143
*BMI*	ADC	2.450	0.430	0.098
Genotype*BMI	ADC	2.266	0.411	0.118
**b. obesity-associated network**	**Parameter**	*F*	***η*^2^^‡^**	**Sig**.
*Genotype*	FA	0.854	0.610	0.612
*BMI*	FA	0.970	0.640	0.545
Genotype*BMI	FA	0.990	0.645	0.534
*Genotype*	ADC	0.653	0.545	0.745
*BMI*	ADC	1.573	0.742	0.300
Genotype*BMI	ADC	0.666	0.550	0.736

η^2^ = partial eta squared. ^‡^Small effect size: 0.10 ≤ η^2^ < 0.3; medium effect size: 0.3 ≤ η^*2*^ < 0.5; large effect size: η^2^ ≥ 0.5. *Significant with p < 0.05.

Genotype, BMI and their interaction between them had no effect on the ADC of the “risk” or “obesity-associated” WM tracts. No effect on the ADC of the forceps major was found either (Table 3).

### Association between the Neuropsychological Scores and “Risk Tracts” Diffusivity

**Figure 3:**
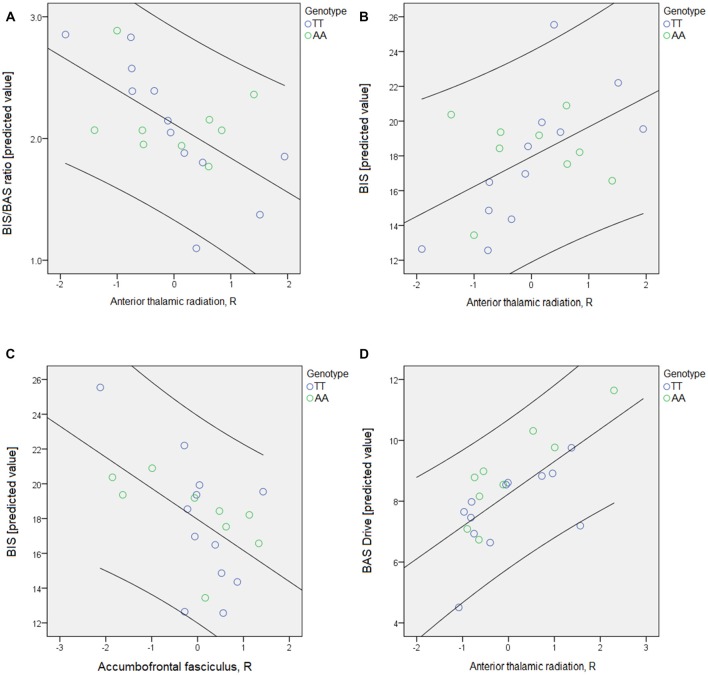
The psychological scores were tested for associations with genotype and the fractional anisotropy (FA) and apparent diffusion coefficient (ADC) of the tracts included in each network through a series of univariate analyses of variance models. For the BIS/BAS questionnaire, the four subscales and the BIS/BAS ratio (BBr) were tested in five different models. The threshold for significance was set at *p* < 0.01, to allow for multiple testing correction according to Bonferroni’s approach. Age was again entered as nuisance covariate. In each model, the white matter (WM) tracts comprising the network of interest were entered as independent variables. Only the “risk-network” results are represented here. **(A)** The predicted value of the BBr is plotted against the FA of the right anterior thalamic radiations (ATR); **(B,C)** the predicted value of the BIS is plotted against the FA of the right ATR **(B)** and of the right accumbofrontal fasciculus **(C)**; **(D)** the predicted value of the BAS Drive subscale is plotted against the ADC of the right ATR. Details regarding the results can be found in Table 4.

When the BIS/BAS questionnaire scores were tested against “risk-tracts” and genotype, the FA of the right ATR significantly predicted the BBr (*p* < 0.002) and BIS (*p* < 0.000) scores (Table 4, Figure 3). The FA of the right accumbofrontal fasciculus predicted the BIS (*p* < 0.009). The Drive subscale of the BAS was found to be significantly associated with the ADC of the left ATR (*p* < 0.01; Table 4, Figure 3). The association between the ADC in the right ATR and the BIS subscale approached significance (*p* < 0.05, uncorrected).

**Table 4:** Significant correlations between the neuropsychological scores, genotype and the “risk-network” WM tracts.

Score	WM tract	*F*	*η*^2^^‡^	Sig.
*FRACTIONAL ANISOTROPY*		
BIS/BAS ratio	Right ATR	15.0	0.518	0.002**
BIS	Right ATR	35.3	0.716	4 × 10^−5^**
	Right Fronto-Accumbens	9.2	0.396	0.009**
*APPARENT DIFFUSION COEFFICIENT*
BAS DRIVE	Left ATR	17.1	0.386	0.010**
BIS	Right ATR	5.0	0.280	0.035

η^2^ = partial eta squared. ^‡^Small effect size: 0.10 ≤ η^2^ < 0.3; medium effect size: 0.3 ≤ η^2^ < 0.5; large effect size: η^2^ ≥ 0.5. **Significant after correction for multiple comparisons.

### Association between the Neuropsychological Scores and “Obesity-Associated Tracts” Diffusivity

When testing the BIS/BAS scores, the BBr was significantly associated with the FA of the left (*p* < 0.003) and right (*p* < 0.005) hippocampal part of the cingulum, and approached significance for several other WM tracts (Table 5). Moreover, the genotype approached significance for an association with the BIS score (*p* < 0.04, uncorrected). No associations were found between the ADC of the “obesity-associated tracts” and the BIS/BAS scores or genotype.

**Table 5:** Significant correlations between the neuropsychological scores, genotype and the “obesity-associated network” WM tracts.

Score	WM tract	*F*	*η*^2‡^	Sig.
FRACTIONAL ANISOTROPY
BIS/BAS RATIO	Genotype	9.2	0.568	0.019
	Left CGH	20.3	0.743	0.003**
	Right CGH	16.2	0.669	0.005**
	Right Cingulate Gyrus	9.9	0.586	0.016
	Left temporal SLF	6.1	0.464	0.043
	Right temporal SLF	8.4	0.546	0.023
BIS	Genotype	5.7	0.449	0.048
	Left CGH	8.9	0.561	0.020

η^2^ = partial eta squared. ^‡^Small effect size: 0.10 ≤ η^*2*^ < 0.3; medium effect size: 0.3 ≤ η^2^ < 0.5; large effect size: η^2^ ≥ 0.5. **Significant after correction for multiple comparisons.

## Discussion

We hypothesized that WM microstructure in specific regions of the reward network would be altered in individuals with genetic risk for obesity despite most of the participants had not yet developed obesity, reflecting the presence of a microstructural damage already at preclinical stages. We have thus tested WM integrity in 21 male participants genotyped on the *FTO* risk SNP rs9939609, through a deterministic tractography analysis. We have detected an effect of genotype, as well as an interaction effect of genotype and BMI, on the WM FA in the “risk tracts” (ATR and accumbofrontal fasciculus), but not in the “obesity-associated” tracts. Our results may suggest that an alteration of the WM connectivity within the central region (basal ganglia) of the brain is already present at subclinical stage in carriers of FTO-risk allele for obesity.

The accumbofrontal fasciculus and the ATR are part of a corticostriatothalamocortical loop (Rigoard et al., 2011), which has been previously reported to be disrupted in obesity (Marqués-Iturria et al., 2015; Kullmann et al., 2016; Papageorgiou et al., 2017). The thalamus WM connectivity, in particular, has been found to be altered in early-life obesity (Ou et al., 2015), and it has been suggested as a potential imaging predictor of BMI in obese people (Park et al., 2015). The accumbofrontal fasciculus, on the other hand, undergoes remarkable structural changes during the life-span, with patterns consistent with developmental models of decision-making (Karlsgodt et al., 2015). The GM volume of the nucleus accumbens has been reported to be lower in the at-risk AA carriers of the *FTO* SNP rs9939609 (de Groot et al., 2015). Moreover, the expression of the serotoninergic receptor 5-HT4 in nucleus accumbens has been reported to be upregulated in obesity, and the level of expression correlated with BMI (Iozzo, 2015). Due to its key role in reward and punishment (Dehkordi et al., 2017; Shin et al., 2017), the nucleus accumbens has been suggested as a target for deep brain stimulation for obesity treatment (Nangunoori et al., 2016).

Only two studies have so far investigated whether brain structural connectivity might be damaged in people with genetic risk for obesity (Dennis et al., 2014; Spieker et al., 2015). Dennis et al. (2014) reported an association of WM integrity in several brain structures with only one gene (NEGR1) out of the 14 obesity-gene selected. While the *FTO* SNP rs9939609 was not included in their analysis, other two *FTO* SNPs were considered (Dennis et al., 2014). Spieker et al. (2015) on the other hand, reported a shared genetic variance between obesity and WM integrity in 10 WM tracts selected *a priori*. The accumbo-frontal fasciculus was not included in this study. Moreover, they used a heritability analysis, where heritability is the proportion of total phenotypic variance that is explained by additive genetic factors (Spieker et al., 2015). Thus, no information regarding the specific genes involved was provided. One study investigating WM hyperintensities, reflective of WM vascular damage, failed to demonstrate any association between the *FTO* gene and WM burden (Ho et al., 2010). However, this study was carried out in elderly participants (with a mean age of 76 years; Ho et al., 2010), when WM vascular damage is frequently observed in the general population (Yoshita et al., 2006; Sachdev et al., 2007).

We suggest that a very close relationship between genetic background, neurovascular-coupling and WM disruption in regional connectivity of central regions of the brain might act together as a predisposing factor for the development of obesity. Neurovascular coupling is in fact associated with neuroinflammatory processes and WM integrity (Soto et al., 2015; Wilhelm et al., 2017). Central reward-related regions within basal ganglia such as thalamus and the nucleus accumbens are particularly vulnerable to pressure dysregulation (Moody et al., 1990; Perles-Barbacaru et al., 2011; Iozzo, 2015). The thalamus and the basal ganglia, in fact, have a peculiar vascular organization, characterized by long arterioles and long muscular arteries, supplied by adjacent sources at the base of the brain (Moody et al., 1990). The terminal arterioles, often narrow and short, do not interdigitate with the terminal arterioles of adjacent territories, posing a greater risk for hypoperfusion and anoxia in these regions compared to other brain territories (Moody et al., 1990). The nucleus accumbens in particular has been reported to be more vulnerable to pressure dysregulation compared to the other regions (Perles-Barbacaru et al., 2011), mainly because of the local expression of dopamingergic receptors (Frank, 2015). Dopamine plays a key role in neurovascular coupling and pressure regulation (Perles-Barbacaru et al., 2011). The reward network is enriched in dopamine receptors (Choi et al., 2006), and a disruption of dopaminergic signaling, particularly through an hyposensitivity of receptors D2 and D3, has been reported in obesity (Frank, 2015). The activity of the receptors D2 and D3 is also regulated by the *FTO* gene (Hess et al., 2013; Sevgi et al., 2015; Heni et al., 2016). Hence, it seems reasonable to think that some of the increased dietary intake and unhealthy eating behaviors identified in carrier of the *FTO* risk variants (Brunkwall et al., 2013) might be associated to an early dysfunction of the WM connectivity of these regions due to a vascular damage on genetic based risk.

We found an effect of the *FTO* risk allele on the FA, rather than the ADC, of the “risk-tracts”. This discrepancy might be due to the different sensitivity that ADC and FA have toward different neural maturation processes, such as fiber organization, myelination, and proliferation and maturation of glial cell bodies and intracellular compartments (Provenzale et al., 2010), particularly during neurogenesis. Indeed, ADC and FA do not necessarily correlate with each other (Provenzale et al., 2010; Leong et al., 2015), and the relationship between ADC and FA values varies for different WM tracts (Provenzale et al., 2010). The FTO gene has been reported to be involved in neurogenesis in mice (Li et al., 2017). Though the underlying mechanisms are still unclear, it is therefore likely that it might affect differently the FA and the ADC of WM tracts.

Future longitudinal studies will be needed to investigate whether WM microstructural changes in specific regions of the reward network can indeed be regarded as a first step in the development of obesity in carrier of the *FTO* at-risk allele. Moreover, our sample was small, and included only male participants, calling for other studies with larger and more heterogeneous cohorts, possibly including also heterozygous *FTO* AT allele carriers, to verify our findings. Finally, we used a deterministic tractography approach with manually defined ROIs for tracts definition. The ROIs were carefully defined according to previous literature and a blind check was carried out by a second operator, however automated techniques and atlas ROIs might be used to avoid any potential bias in ROIs design.

## Conclusion

We report an alteration of WM microstructure in the accumbofrontal fasciculi and ATR of males with the AA at-risk allele of the *FTO* SNP rs9939609, compared to the non-risk allele TT. We suggest that ATR and accumbofrontal fasciculi damage might be a predisposing factor for obesity at brain level in the AA allele carriers. Such damage might derive from the interplay between genetic background, altered neurovascular-coupling and the high susceptibility of the thalamus and nucleus accumbens to vascular damage, due to their peculiar anatomo-vascular characteristics. The disruption in dopaminergic signaling, caused by changes in expression and sensitivity of dopaminergic receptors induced by the risk-allele, might in fact lead to an altered neurovascular coupling in the reward network, which in turn might contribute in determining WM microstructural damage in highly vulnerable regions such the thalamus and nucleus accumbens.

## Author Contributions

GO performed the analyses, contributed to the interpretation of the data and wrote the article; FL contributed to the interpretation of data and wrote the article; LW contributed to the interpretation of the data; E-ML directed imaging procedures and screened the scans for safety; HBS was involved in the initial design of the study, oversaw the project and was involved in planning/organization of the experimental procedures. All authors revised the article and gave final approval of the version to be published.

## Conflict of Interest Statement

The authors declare that the research was conducted in the absence of any commercial or financial relationships that could be construed as a potential conflict of interest.

## Footnotes

^1^http://www.genpat.uu.se/node462

^2^http://trackvis.org/dtk/

^3^http://trackvis.org/

^4^https://www.ibm.com/us-en/marketplace/spss-statistics

## References

[B1] Alexander A. L. Lee J. E. Lazar M. Field A. S. (2007). Diffusion tensor imaging of the brain. Neurotherapeutics 4, 316–329. 10.1016/j.nurt.2007.05.011 17599699 PMC2041910

[B65] Blechert J. Klackl J. Miedl S. F. Wilhelm F. H. (2016). To eat or not to eat: effects of food availability on reward system activity during food picture viewing. Appetite 99, 254–261. 10.1016/j.appet.2016.01.006 26796027

[B2] Bracht T. Linden D. Keedwell P. (2015). A review of white matter microstructure alterations of pathways of the reward circuit in depression. J. Affect. Disord. 187, 45–53. 10.1016/j.jad.2015.06.041 26318270

[B3] Brunkwall L. Ericson U. Hellstrand S. Gullberg B. Orho-Melander M. Sonestedt E. (2013). Genetic variation in the fat mass and obesity-associated gene (FTO) in association with food preferences in healthy adults. Food Nutr. Res. 57:20028. 10.3402/fnr.v57i0.20028 23589710 PMC3625705

[B4] Camara E. Rodriguez-Fornells A. Ye Z. Münte T. F. (2009). Reward networks in the brain as captured by connectivity measures. Front. Neurosci. 3, 350–362. 10.3389/neuro.01.034.2009 20198152 PMC2796919

[B66] Carnell S. Benson L. Chang K. V. Wang Z. Huo Y. Geliebter A.. (2017). Neural correlates of familial obesity risk and overweight in adolescence. Neuroimage 159, 236–247. 10.1016/j.neuroimage.2017.07.052 28754348 PMC5671352

[B5] Carver C. White T. L. (1994). Behavioral inhibition, behavioral activation, and affective responses to impending reward and punishment: the BIS/BAS scales. J. Pers. Soc. Psychol. 67, 319–333. 10.1037//0022-3514.67.2.319

[B6] Cecil J. E. Tavendale R. Watt P. Hetherington M. M. Palmer C. N. (2008). An obesity-associated FTO gene variant and increased energy intake in children. N. Engl. J. Med. 359, 2558–2566. 10.1056/NEJMoa0803839 19073975

[B7] Chapman S. B. Aslan S. Spence J. S. Hart J. J. Jr. Bartz E. K. Didehbani N.. (2015). Neural mechanisms of brain plasticity with complex cognitive training in healthy seniors. Cereb. Cortex 25, 396–405. 10.1093/cercor/bht234 23985135 PMC4351428

[B9] Choi J. K. Chen Y. I. Hamel E. Jenkins B. G. (2006). Brain hemodynamic changes mediated by dopamine receptors: role of the cerebral microvasculature in dopamine-mediated neurovascular coupling. Neuroimage 30, 700–712. 10.1016/j.neuroimage.2005.10.029 16459104

[B8] Cho Y. T. Fromm S. Guyer A. E. Detloff A. Pine D. S. Fudge J. L.. (2013). Nucleus accumbens, thalamus and insula connectivity during incentive anticipation in typical adults and adolescents. Neuroimage 66, 508–521. 10.1016/j.neuroimage.2012.10.013 23069809 PMC3949208

[B10] de Groot C. Felius A. Trompet S. de Craen A. J. Blauw G. J. van Buchem M. A.. (2015). Association of the fat mass and obesity-associated gene risk allele, rs9939609A, and reward-related brain structures. Obesity 23, 2118–2122. 10.1002/oby.21191 26337140

[B11] Dehkordi O. Rose J. E. Dávila-García M. I. Millis R. M. Mirzaei S. A. Manaye K. F.. (2017). Neuroanatomical relationships between orexin/hypocretin-containing neurons/nerve fibers and nicotine-induced c-Fos-activated cells of the reward-addiction neurocircuitry. J. Alcohol. Drug Depend. 5:273. 10.4172/2329-6488.1000273 29038792 PMC5640973

[B12] Dennis E. L. Jahanshad N. Braskie M. N. Warstadt N. M. Hibar D. P. Kohannim O.. (2014). Obesity gene NEGR1 associated with white matter integrity in healthy young adults. Neuroimage 102, 548–557. 10.1016/j.neuroimage.2014.07.041 25072390 PMC4269485

[B15] Frank G. K. (2015). Advances from neuroimaging studies in eating disorders. CNS Spectr. 20, 391–400. 10.1017/s1092852915000012 25902917 PMC4989857

[B16] Frayling T. M. Timpson N. J. Weedon M. N. Zeggini E. Freathy R. M. Lindgren C. M.. (2007). A common variant in the FTO gene is associated with body mass index and predisposes to childhood and adult obesity. Science 316, 889–894. 10.1126/science.1141634 17434869 PMC2646098

[B17] Goodarzi M. O. (2017). Genetics of obesity: what genetic association studies have taught us about the biology of obesity and its complications. Lancet Diabetes Endocrinol. [Epub ahead of print]. 10.1016/s2213-8587(17)30200-0 28919064

[B18] Gupta A. Mayer E. A. Sanmiguel C. P. Van Horn J. D. Woodworth D. Ellingson B. M.. (2015). Patterns of brain structural connectivity differentiate normal weight from overweight subjects. Neuroimage Clin. 7, 506–517. 10.1016/j.nicl.2015.01.005 25737959 PMC4338207

[B19] Heni M. Kullmann S. Ahlqvist E. Wagner R. Machicao F. Staiger H.. (2016). Interaction between the obesity-risk gene FTO and the dopamine D2 receptor gene ANKK1/TaqIA on insulin sensitivity. Diabetologia 59, 2622–2631. 10.1007/s00125-016-4095-0 27600277

[B20] Hess M. E. Hess S. Meyer K. D. Verhagen L. A. Koch L. Brönneke H. S.. (2013). The fat mass and obesity associated gene (Fto) regulates activity of the dopaminergic midbrain circuitry. Nat. Neurosci. 16, 1042–1048. 10.1038/nn.3449 23817550

[B21] Ho A. J. Stein J. L. Hua X. Lee S. Hibar D. P. Leow A. D.. (2010). A commonly carried allele of the obesity-related FTO gene is associated with reduced brain volume in the healthy elderly. Proc. Natl. Acad. Sci. U S A 107, 8404–8409. 10.1073/pnas.0910878107 20404173 PMC2889537

[B22] Iozzo P. (2015). Metabolic imaging in obesity: underlying mechanisms and consequences in the whole body. Ann. N Y Acad. Sci. 1353, 21–40. 10.1111/nyas.12880 26335600

[B23] Jacobsson J. A. Schiöth H. B. Fredriksson R. (2012). The impact of intronic single nucleotide polymorphisms and ethnic diversity for studies on the obesity gene FTO. Obes. Rev. 13, 1096–1109. 10.1111/j.1467-789x.2012.01025.x 22931202

[B24] Jenkinson M. Beckmann C. F. Behrens T. E. Woolrich M. W. Smith S. M. (2012). Fsl. Neuroimage 62, 782–790. 10.1016/j.neuroimage.2011.09.015 21979382

[B25] Karlsgodt K. H. John M. Ikuta T. Rigoard P. Peters B. D. Derosse P.. (2015). The accumbofrontal tract: diffusion tensor imaging characterization and developmental change from childhood to adulthood. Hum. Brain Mapp. 36, 4954–4963. 10.1002/hbm.22989 26366528 PMC4715564

[B26] Kullmann S. Callaghan M. F. Heni M. Weiskopf N. Scheffler K. Häring H. U.. (2016). Specific white matter tissue microstructure changes associated with obesity. Neuroimage 125, 36–44. 10.1016/j.neuroimage.2015.10.006 26458514 PMC4692452

[B27] Leong D. Calabrese E. White L. E. Wei P. Chen S. Platt S. R.. (2015). Correlation of diffusion tensor imaging parameters in the canine brain. Neuroradiol. J. 28, 12–18. 10.15274/NRJ-2014-10110 25924167 PMC4757115

[B28] Li L. Zang L. Zhang F. Chen J. Shen H. Shu L.. (2017). Fat mass and obesity-associated (FTO) protein regulates adult neurogenesis. Hum. Mol. Genet. 26, 2398–2411. 10.1093/hmg/ddx128 28398475 PMC6192412

[B29] Loos R. J. Yeo G. S. (2014). The bigger picture of FTO: the first GWAS-identified obesity gene. Nat. Rev. Endocrinol. 10, 51–61. 10.1038/nrendo.2013.227 24247219 PMC4188449

[B30] Marqués-Iturria I. Scholtens L. H. Garolera M. Pueyo R. García-García I. González-Tartiere P.. (2015). Affected connectivity organization of the reward system structure in obesity. Neuroimage 111, 100–106. 10.1016/j.neuroimage.2015.02.012 25687594

[B67] Michaud A. Vainik U. Garcia-Garcia I. Dagher A. (2017). Overlapping neural endophenotypes in addiction and obesity. Front. Endocrinol. 8:127. 10.3389/fendo.2017.00127 28659866 PMC5469912

[B32] Moody D. M. Bell M. A. Challa V. R. (1990). Features of the cerebral vascular pattern that predict vulnerability to perfusion or oxygenation deficiency: an anatomic study. Proc. Natl. Acad. Sci. U S A 11, 431–439. 2112304 PMC8367475

[B34] Nangunoori R. K. Tomycz N. D. Oh M. Y. Whiting D. M. (2016). Deep brain stimulation for obesity: from a theoretical framework to practical application. Neural Plast. 2016:7971460. 10.1155/2016/7971460 26819774 PMC4706960

[B35] Ou X. Andres A. Pivik R. T. Cleves M. A. Badger T. M. (2015). Brain gray and white matter differences in healthy normal weight and obese children. J. Magn. Reson. Imaging 42, 1205–1213. 10.1002/jmri.24912 25865707

[B36] Papageorgiou I. Astrakas L. G. Xydis V. Alexiou G. A. Bargiotas P. Tzarouchi L.. (2017). Abnormalities of brain neural circuits related to obesity: a Diffusion Tensor Imaging study. Magn. Reson. Imaging 37, 116–121. 10.1016/j.mri.2016.11.018 27899333

[B37] Park B. Y. Seo J. Yi J. Park H. (2015). Structural and functional brain connectivity of people with obesity and prediction of body mass index using connectivity. PLoS One 10:e0141376. 10.1371/journal.pone.0141376 26536135 PMC4633033

[B38] Perles-Barbacaru T. A. Procissi D. Demyanenko A. V. Hall F. S. Uhl G. R. Jacobs R. E. (2011). Quantitative pharmacologic MRI: mapping the cerebral blood volume response to cocaine in dopamine transporter knockout mice. Neuroimage 55, 622–628. 10.1016/j.neuroimage.2010.12.048 21185387 PMC3035982

[B39] Provenzale J. M. Isaacson J. Chen S. Stinnett S. Liu C. (2010). Correlation of apparent diffusion coefficient and fractional anisotropy values in the developing infant brain. Am. J. Roentgenol. 195, W456–W462. 10.2214/AJR.10.4886 21098179 PMC3640803

[B40] Rigoard P. Buffenoir K. Jaafari N. Giot J. P. Houeto J. L. Mertens P.. (2011). The accumbofrontal fasciculus in the human brain: a microsurgical anatomical study. Neurosurgery 68, 1102–1111; discussion 1111. 10.1227/NEU.0b013e3182098e48 21242843

[B41] Sachdev P. Wen W. Chen X. Brodaty H. (2007). Progression of white matter hyperintensities in elderly individuals over 3 years. Neurology 68, 214–222. 10.1212/01.WNL.0000251302.55202.73 17224576

[B42] Sällman Almén M. Rask-Andersen M. Jacobsson J. A. Ameur A. Kalnina I. Moschonis G.. (2013). Determination of the obesity-associated gene variants within the entire FTO gene by ultra-deep targeted sequencing in obese and lean children. Int. J. Obes. 37, 424–431. 10.1038/ijo.2012.57 22531089 PMC3595467

[B43] Sesack S. R. Grace A. A. (2010). Cortico-Basal Ganglia reward network: microcircuitry. Neuropsychopharmacology 35, 27–47. 10.1038/npp.2009.93 19675534 PMC2879005

[B44] Sevgi M. Rigoux L. Kuhn A. B. Mauer J. Schilbach L. Hess M. E.. (2015). An obesity-predisposing variant of the FTO gene regulates D2R-dependent reward learning. J. Neurosci. 35, 12584–12592. 10.1523/JNEUROSCI.1589-15.2015 26354923 PMC6605390

[B45] Shin J. H. Adrover M. F. Alvarez V. A. (2017). Distinctive modulation of dopamine release in the nucleus accumbens shell mediated by dopamine and acetylcholine receptors. J. Neurosci. 37, 11166–11180. 10.1523/jneurosci.0596-17.2017 29030431 PMC5688525

[B46] Smith S. M. (2002). Fast robust automated brain extraction. Hum. Brain Mapp. 17, 143–155. 10.1002/hbm.10062 12391568 PMC6871816

[B47] Soares J. M. Marques P. Alves V. Sousa N. (2013). A hitchhiker’s guide to diffusion tensor imaging. Front. Neurosci. 7:31. 10.3389/fnins.2013.00031 23486659 PMC3594764

[B48] Sorond F. A. Hurwitz S. Salat D. H. Greve D. N. Fisher N. D. (2013). Neurovascular coupling, cerebral white matter integrity and response to cocoa in older people. Neurology 81, 904–909. 10.1212/wnl.0b013e3182a351aa 23925758 PMC3885215

[B49] Soto I. Graham L. C. Richter H. J. Simeone S. N. Radell J. E. Grabowska W.. (2015). APOE stabilization by exercise prevents aging neurovascular dysfunction and complement induction. PLoS Biol. 13:e1002279. 10.1371/journal.pbio.1002279 26512759 PMC4626092

[B50] Speakman J. R. Rance K. A. Johnstone A. M. (2008). Polymorphisms of the FTO gene are associated with variation in energy intake, but not energy expenditure. Obesity 16, 1961–1965. 10.1038/oby.2008.318 18551109

[B51] Spieker E. A. Kochunov P. Rowland L. M. Sprooten E. Winkler A. M. Olvera R. L.. (2015). Shared genetic variance between obesity and white matter integrity in Mexican Americans. Front. Genet. 6:26. 10.3389/fgene.2015.00026 25763009 PMC4327744

[B68] Verdejo-Román J. Vilar-López R. Navas J. F. Soriano-Mas C. Verdejo-García A. (2017). Brain reward system’s alterations in response to food and monetary stimuli in overweight and obese individuals. Hum. Brain Mapp. 38, 666–667. 10.1002/hbm.23407 27659185 PMC6867019

[B52] Vergani F. Martino J. Morris C. Attems J. Ashkan K. Dell’Acqua F. (2016). Anatomic connections of the subgenual cingulate region. Neurosurgery 79, 465–472. 10.1227/neu.0000000000001315 27327267

[B53] Voigt D. C. Dillard J. P. Braddok K. H. Anderson J. W. Sopori P. Stephenson M. P. (2009). Carver and White’s (1994) BIS/BAS scales and their relationship to risky health behaviours. Pers. Individ. Dif. 47, 89–93. 10.1016/j.paid.2009.02.003

[B55] Wakana S. Caprihan A. Panzenboeck M. M. Fallon J. H. Perry M. Gollub R. L.. (2007). Reproducibility of quantitative tractography methods applied to cerebral white matter. Neuroimage 36, 630–644. 10.1016/j.neuroimage.2007.02.049 17481925 PMC2350213

[B56] Wilhelm I. Nyúl-Tóth Á. Kozma M. Farkas A. E. Krizbai I. A. (2017). Role of pattern recognition receptors of the neurovascular unit in inflamm-aging. Am. J. Physiol. Heart Circ. Physiol. 313, H1000–H1012. 10.1152/ajpheart.00106.2017 28801521

[B57] Xu J. Kober H. Carroll K. M. Rounsaville B. J. Pearlson G. D. Potenza M. N. (2012). White matter integrity and behavioral activation in healthy subjects. Hum. Brain Mapp. 33, 994–1002. 10.1002/hbm.21275 21618658 PMC3169726

[B58] Yager L. M. Garcia A. F. Wunsch A. M. Ferguson S. M. (2015). The ins and outs of the striatum: role in drug addiction. Neuroscience 301, 529–541. 10.1016/j.neuroscience.2015.06.033 26116518 PMC4523218

[B59] Yang J. Loos R. J. Powell J. E. Medland S. E. Speliotes E. K. Chasman D. I.. (2012). FTO genotype is associated with phenotypic variability of body mass index. Nature 490, 267–272. 10.1038/nature11401 22982992 PMC3564953

[B60] Yang X. H. Wang Y. Wang D. F. Tian K. Cheung E. F. C. Xie G. R.. (2017). White matter microstructural abnormalities and their association with anticipatory anhedonia in depression. Psychiatry Res. 264, 29–34. 10.1016/j.pscychresns.2017.04.005 28437669

[B61] Yoshita M. Fletcher E. Harvey D. Ortega M. Martinez O. Mungas D. M.. (2006). Extent and distribution of white matter hyperintensities in normal aging, MCI, and AD. Neurology 67, 2192–2198. 10.1212/01.wnl.0000249119.95747.1f 17190943 PMC3776588

